# Comparative Analysis of Hydrosol Volatile Components of *Citrus × Aurantium* ‘Daidai’ and *Citrus × Aurantium* L. Dried Buds with Different Extraction Processes Using Headspace-Solid-Phase Microextraction with Gas Chromatography–Mass Spectrometry

**DOI:** 10.3390/molecules29153498

**Published:** 2024-07-26

**Authors:** Xinyue Xie, Huiling Xue, Baoshan Ma, Xiaoqian Guo, Yanli Xia, Yuxia Yang, Ke Xu, Ting Li, Xia Luo

**Affiliations:** 1 College of Food and Biological Engineering, Chengdu University, Chengdu 610106, China; x18783809950@163.com (X.X.); findlot@sohu.com (H.X.); baoshanqm@163.com (B.M.); ank29596@foxmail.com (X.G.); 18328184283@163.com (T.L.); 2 Sichuan Academy of Chinese Medicine Sciences, Chengdu 610041, China; lx287748567@163.com; 3 Sichuan Provincial Horticultural Crop Technology Extension Station, Chengdu 610041, China; ranafood@outlook.com

**Keywords:** *Citrus × aurantium* ‘Daidai’, *Citrus × aurantium* L., hydrosol, extraction process, HS-SPME-GC-MS

## Abstract

This work used headspace solid-phase microextraction with gas chromatography–mass spectrometry (HS-SPME-GC–MS) to analyze the volatile components of hydrosols of *Citrus × aurantium* ‘Daidai’ and *Citrus × aurantium* L. dried buds (CAVAs and CADBs) by immersion and ultrasound–microwave synergistic-assisted steam distillation. The results show that a total of 106 volatiles were detected in hydrosols, mainly alcohols, alkenes, and esters, and the high content components of hydrosols were linalool, α-terpineol, and trans-geraniol. In terms of variety, the total and unique components of CAVA hydrosols were much higher than those of CADB hydrosols; the relative contents of 13 components of CAVA hydrosols were greater than those of CADB hydrosols, with geranyl acetate up to 15-fold; all hydrosols had a citrus, floral, and woody aroma. From the pretreatment, more volatile components were retained in the immersion; the relative contents of linalool and α-terpineol were increased by the ultrasound–microwave procedure; and the ultrasound–microwave procedure was favorable for the stimulation of the aroma of CAVA hydrosols, but it diminished the aroma of the CADB hydrosols. This study provides theoretical support for in-depth exploration based on the medicine food homology properties of CAVA and for improving the utilization rate of waste resources.

## 1. Introduction

According to the Chinese Pharmacopoeia [1], *Aurantii Fructus* [2] is the dried immature fruit of *Citrus × aurantium* L. (also known as bitter orange) and its cultivated varieties, which is a *Citrus × aurantium* L. variety of the citrus plant of the family Rutaceae and is known for its anti-inflammatory, antioxidant, antitumor, and immunomodulatory activities due to its rich content of flavonoids, such as neohesperidin and naringin, and alkaloids, such as synephrine and N-methyltyramine [3,4], and so on. It is a traditional bulk Chinese herbal medicine and has been widely used in both traditional Chinese and modern medicine [5]. *Citrus × aurantium* ‘Daidai’ is one of the main cultivated varieties of *Citrus × aurantium* L., and its dried buds are called *Citrus × aurantium* L. var. *amara* Engl. (CAVAs) [6,7]. It is the only one of the several pharmacopoeia-licensed varieties of *Citrus × aurantium* L. that has entered into the national catalog of medicine food homology and has received wide attention, and a great number of studies have reported on the constituents and pharmacological effects of CAVAs. Studies have shown that CAVAs contain flavonoids, volatile oils, coumarins, and other components, with anti-inflammatory, antitumor, antioxidant, antibacterial, antiviral, hypolipidemic, and other pharmacological effects, and are mainly used for the treatment of symptoms such as food accumulation, phlegm, chest and abdominal stuffiness, pain, oligophagia, nausea, and vomiting, and so on [8,9,10,11,12]. At present, the dried buds of *Citrus × aurantium* L. (CADBs) are mainly used as a source of natural flavor, and there is a lack of systematic research on its composition and pharmacological effects; therefore, it cannot be used as a homologous component in medicine and food.

*Citrus × aurantium* L. has a long history of cultivation in China and has been planted in Jiangsu, Zhejiang, Fujian, Sichuan, and Guizhou [13]. Its flowers are often used to extract essential oils, and the quality of its essential oils is influenced by the extraction process. The yield and composition of essential oils may vary to some extent depending on the extraction method used. Some commonly employed methods for extracting essential oils include steam distillation, supercritical CO_2_ extraction [9], solvent extraction, etc. Steam distillation is often favored due to its affordability and simplicity, despite drawbacks such as lengthy extraction time and low efficiency. To optimize the advantages, it is common practice to employ pretreatments like immersion, ultrasound, and microwave procedure on the materials. Research has demonstrated that immersing a component for a specific duration of time can result in the dissolution of some active components without altering their properties. Additionally, ultrasound cavitation can induce the rupture of plant cell walls, leading to an increased dissolution of active components. Moreover, microwave energy can cause the rapid vaporization of water within the cells, resulting in elevated local temperature and pressure, thereby facilitating the dissolution of more active components [14,15,16].

When flowers of *Citrus × aurantium* L. are used to extract essential oils, one by-product will be produced, i.e., hydrosol, which contains a large number of volatile components with certain activities, but only the essential oil is usually retained in the production, and the hydrosol is not valued, which is a waste of resources; at the same time, the type and content of the hydrosol components vary according to different essential oil extraction processes. There is no relevant report on this subject at present, and the quality is not known.

In order to compare the quality differences between the medicinal and food homologous CAVAs and common CADBs from various perspectives, and with a view of providing theoretical support for the in-depth exploration of the medicinal and food homologous basis of CAVAs as well as to improve the utilization rate of waste resources, CAVAs and CADBs were subjected to two pretreatments, namely the immersion and ultrasound–microwave procedures, and were subjected to extraction by steam distillation to collect the hydrosols. The volatile components in the hydrosol were enriched and characterized by HS-SPME-GC-MS (an advanced technique widely used for the qualitative and quantitative analyses of volatile constituents, with the advantages of being more rapid, sensitive, efficient, and accurate) [17], with the goal of filling the gaps in the relevant research, enhancing the utilization of resources, reducing the wastage of resources, and ultimately laying the theoretical foundations for the in-depth and intensive processing of hydrosols.

## 2. Results and Discussion

### 2.1. Analysis of Volatile Components of CAVA IH and CAVA UH

The HS-SPME-GC-MS method was used to determine the volatile components of CAVA IH, and the relevant information is presented in (Table 1).

The peak times of the main components of CAVA IH ranged from 6.00 min to 32.00 min. A total of 67 compounds were detected by HS-SPME-GC-MS, including 19 alcohols, relative content of about 63.12%; 20 alkenes, with a relative content of about 8.48%; 7 esters, relative content of about 4.57%; 5 aromatic hydrocarbons, relative content of about 0.72%; 7 alkanes, relative content of about 0.47%; 3 ketones, relative content of about 0.25%; 1 phenolic, relative content of about 0.12%; 1 aldehyde, relative content of about 0.03%; 1 ether, relative content of about 0.01%; 1 indene, relative content of about 0.01%; and 2 other compounds, relative content of about 1.05%. The number of alcohol, alkene, and ester kinds accounted for about 58.21%, and the total content comprised about 76.17%, among which the main component linalool (1.5674 mg/kg, 36.79%) ranked first, α-terpineol (0.4013 mg/kg, 9.42%) ranked second, and trans-geraniol (0.3119 mg/kg, 7.32%) ranked third, followed in order by spathulenol (0.1937 mg/kg, 4.55%), geraniol ester (0.1066 mg/kg, 2.50%), d-limonene (0.0937 mg/kg, 2.20%), nerol (0.0841 mg/kg, 1.97%), β-myrcene (0.0769 mg/kg, 1.80%), neryl acetate (0.0647 mg/kg, 1.52%), and caryophyllene oxide (0.0426 mg/kg, 1.00%). The relative content of the remaining components was less than 1%. 

The HS-SPME-GC-MS method was used to determine the volatile components of CAVA UH, and the relevant information is presented in (Table 2).

The peak times of the main components of CAVA UH ranged from 6.00 min to 32.00 min. A total of 54 compounds were detected by HS-SPME-GC-MS, including 12 alcohols, relative content of about 69.87%; 14 alkenes, with a relative content of about 7.26%; 9 esters, relative content of about 3.69%; 6 alkanes, relative content of about 0.27%; 3 ketones, relative content of about 0.38%; 2 aromatic hydrocarbons, relative content of about 0.32%; 2 acids, relative content of about of 0.19%; 1 phenolic, relative content of about 0.17%; 2 aldehydes, relative content of about 0.08%; 1 phenylhydrazone, relative content of about 0.07%; and 2 other compounds, relative content of about 0.06%. The number of alcohol, alkene, and ester kinds accounted for about 64.81%, and the total content comprised about 80.82%, among which the main component linalool (2.0741 mg/kg, 44.38%) ranked first, α-terpineol (0.5052 mg/kg, 10.81%) ranked second, and trans-geraniol (0.3413 mg/kg, 7.30%) ranked third, followed in order by spathulenol (0.1626 mg/kg, 3.48%), d-limonene (0.1025 mg/kg, 2.19%), nerol (0.0914 mg/kg, 1.96%), acetic acid, geraniol ester (0.0909 mg/kg, 1.94%), β-myrcene (0.0810 mg/kg, 1.73%), and neryl acetate (0.0597 mg/kg, 1.28%). The relative content of the remaining components was less than 1%.

### 2.2. Analysis of Volatile Components of CADB IH and CADB UH

The HS-SPME-GC-MS method was used to determine the volatile components of CADB IH, and the relevant information is presented in (Table 3).

The peak times of the main components of CADB IH were in the range of 11.00 min to 28.00 min. A total of 32 compounds were detected by HS-SPME-GC-MS, including 12 alcohols, with a relative content of about 83.00%; 9 alkenes, relative content of about 2.67%; 4 esters, relative content of about 0.45%; 1 phenolic, relative content of about 0.13%; 3 ketones, relative content of about 0.12%; 1 cycloalkane hydrocarbon, relative content of about 0.07%; 1 aromatic hydrocarbon, relative content of about 0.03%; and 1 alkane, relative content of about 0.02%. The number of alcohol, alkene, and ester kinds accounted for about 78.13%, and the total content comprised about 86.12%, among which the main component linalool (3.4712 mg/kg, 56.54%) ranked first, α-terpineol (0.7361 mg/kg, 11.99%) ranked second, and trans-geraniol (0.4865 mg/kg, 7.92%) ranked third, followed in order by nerol (0.2149 mg/kg, 3.50%) and spathulenol (0.1041 mg/kg, 1.70%). The relative content of the remaining components was less than 1%.

The HS-SPME-GC-MS method was used to determine the volatile components of CADB UH, and the relevant information is presented in (Table 4).

The peak times of the main components of CADB UH were in the range of 11.00 min to 28.00 min. A total of 26 compounds were detected by HS-SPME-GC-MS, including 14 alcohols, with a relative content of about 83.02%; 5 alkenes, relative content of about 1.74%; 3 esters, relative content of about 0.29%; 2 ketones, relative content of about 0.22%; 1 phenolic, relative content of about 0.12%; and 1 alkane, relative content of about 0.03%. The number of alcohol, alkene, and ester kinds accounted for about 84.62%, and the total content comprised about 85.05%, among which the main component linalool (3.1096 mg/kg, 56.44%) ranked first, α-terpineol (0.7186 mg/kg, 13.04%) ranked second, and trans-geraniol (0.3591 mg/kg, 6.52%) ranked third, followed in order by nerol (0.1795 mg/kg, 3.26%) and spathulenol (0.1147 mg/kg, 2.08%). The relative content of the remaining components was less than 1%.

The above results were similar to the previous study, which found that the main constituents of *Citrus × aurantium* L. flower hydrosols were linalool, α-terpineol, geraniol [18], nerol, linalyl acetate, d-limonene, and neryl acetate [19]. According to the actual test results, variations in the retention times of certain components were observed, which can be attributed to slight deviations in the heating protocols and the complex compositions of the components.

### 2.3. Comprehensive Analysis of Volatile Components of CAVA Hydrosols and CADB Hydrosols

**Figure 1:**
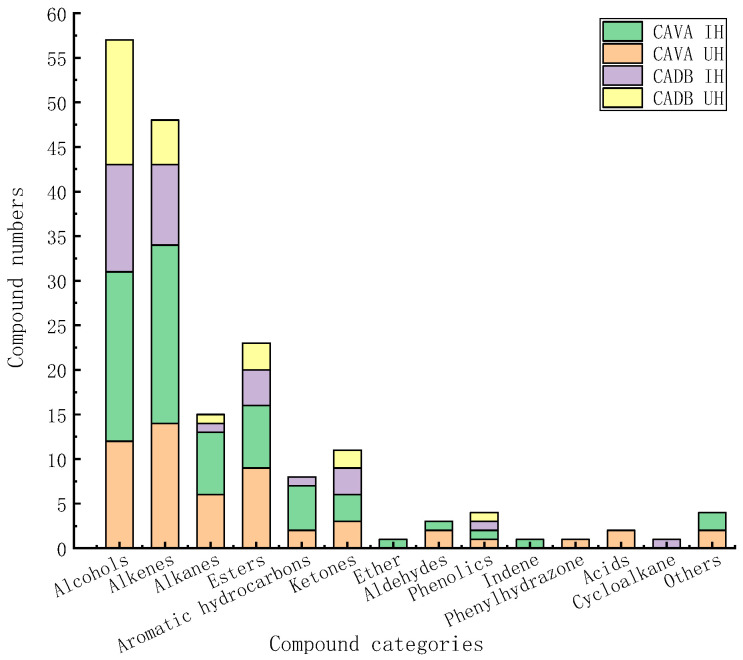
Comparison of the amounts of volatile components obtained in CAVA hydrosols and CADB hydrosols.

**Figure 2:**
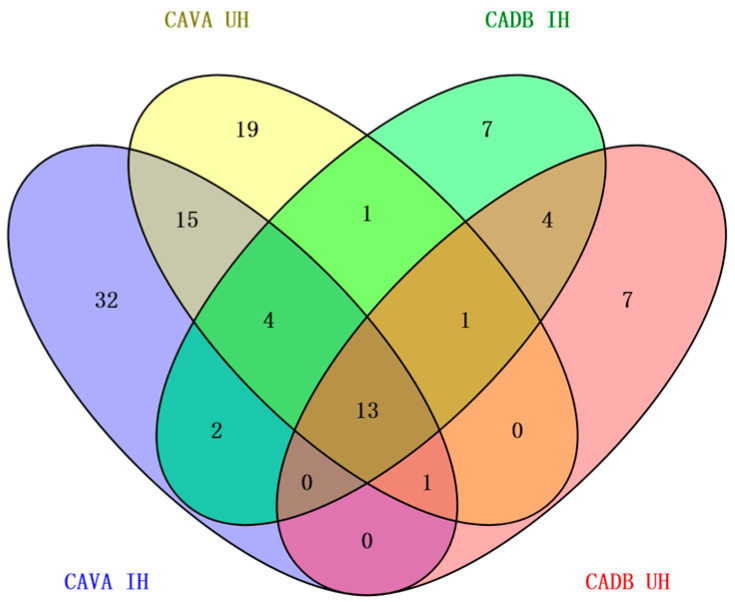
Venn diagram of volatile components obtained in CAVA hydrosols and CADB hydrosols. Different colors correspond to different hydrosols, consistent with the textual identification on the figure; numbers indicate the number of unique and shared components in the four hydrosols.

As shown in (Figure 1 and Figure 2), 67, 54, 32, and 26 volatile constituents were identified in CAVA IH, CAVA UH, CADB IH, and CADB UH, respectively, after eliminating duplicates, totaling 106 compounds. Alcohols, alkenes, and esters were the main constituents, constituting approximately 58.21%, 66.67%, 78.13%, and 84.62% of the total types of components in the four hydrosols. This marked a significant increase in compound diversity in the four hydrosols compared to previous studies [16,20,21,22,23] (20–60 kinds), demonstrating a noteworthy growth in the number of detected compounds in lime flowers.

Combined with (Figure 2) and (Table 5), the total components of CAVA hydrosols (67, 54) exceeded those of CADB hydrosols (32, 26). The total components of immersion (67, 32) outweighed those of the ultrasound–microwave procedure (54, 26); CAVA hydrosols and CADB hydrosols had 22 components in common, of which 13 were common to all four types of hydrosols; CAVA hydrosols contained a significantly higher number of unique components (66) compared to CADB hydrosols (18), while more unique constituents could be obtained from immersion (32, 7) than from the ultrasound–microwave procedure (19, 7).

As shown in (Figure 2), the comparison of various pretreatment methods revealed that the immersion and ultrasound–microwave procedures for CAVA hydrosols exhibited 15 common constituents, whereas CADB hydrosols had only 4. When comparing the different varieties, CAVA hydrosols and CADB hydrosols had two common constituents in immersion, whereas CAVA hydrosols and CADB hydrosols had no common constituents in the ultrasound–microwave procedure. These differences could be attributed to varietal differences [24,25].

When the pretreatments were the same, the CAVA hydrosols had higher total and exclusive components compared to the CADB hydrosols, with fewer common components between them; when the varieties were the same, the immersion contained a greater variety of components compared to the ultrasound–microwave procedure. Furthermore, the CAVA hydrosols contained a greater number of distinct components in the immersion than in the ultrasound–microwave procedure.

### 2.4. Cluster Analysis of Volatile Components of CAVA Hydrosols and CADB Hydrosols

**Figure 3:**
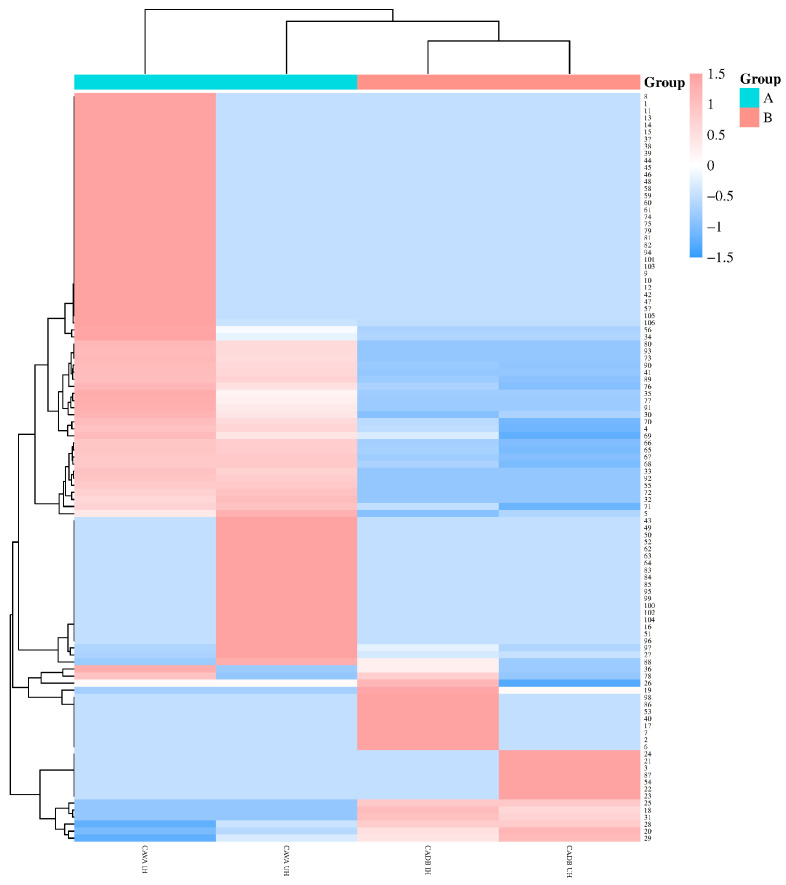
Heatmap analysis of volatile components of hydrosols. In the figure, 1-106 are the hydrosol compound numbers, while the specific names are shown in Table 6 (compounds).

A clustered heatmap is displayed in (Figure 3), with red areas representing higher levels of components and blue areas representing lower levels of components. As can be seen in it, the four hydrosols had less similarity in composition type and content; the volatile components with more prominent content were visualized. With the same pretreatments, there are significant differences in the composition and content of volatile constituents between CAVA hydrosols and CADB hydrosols, and among the common major constituents, the linalool (36.79%/44.38%), α-terpineol (9.42%/10.81%), and nerol (1.97%/1.96%) contents of CAVA IH/CAVA UH were less than those of CADB IH/CADB UH, respectively (56.54%/56.44%; 11.99%/13.04%; 3.50%/3.26%). However, the relative contents of the remaining components found in the CAVA hydrosols had a significant advantage, i.e., based on the same treatments, the relative contents of 13 components such as d-carvone, β-myrcene, d-limonene, neryl acetate, etc., of CAVA hydrosols were greater than those of CADB hydrosols, ranging from 1 to 15-fold. Particularly, neryl acetate and geranyl acetate exhibited the highest increases, reaching up to 9.85 and 15-fold, respectively. These differences could be attributed to the variations in the volatile components’ composition within the respective varieties.

When the varieties were the same, the linalool (44.38%) and α-terpineol (10.81%) contents of the ultrasound–microwave procedure in the CAVA hydrosols were greater than those of the immersion (36.79% and 9.42%, respectively), and the spathulenol (3.68%), geranyl acetate (1.94%), trans-geraniol (7.30%), and caryophyllene oxide (0.04%) contents were lower compared to those from immersion (4.55%, 2.50%, 7.32%, and 1.00%, respectively). The content of α-terpineol (13.04%) in the ultrasound–microwave procedure of CADB hydrosols was greater than that of the immersion (11.99%), whereas the content of trans-geraniol (6.52%) was less than that of the immersion (7.92%).

In summary, it can be seen that the two pretreatments have their unique characteristics. From the standpoint of volatile components, the decrease in components through the ultrasound–microwave procedure as opposed to immersion might stem from the alteration of certain thermally sensitive components with lower content following oxidation and polymerization; according to a report, Lv et al. used three methods to extract CAVA essential oil, traditional hydrodistillation, ultrasound-assisted hydrodistillation, and microwave-assisted hydrodistillation, and identified 30, 33, and 50 compounds, respectively [26]. These results align closely with those of our study, indicating that the ultrasound–microwave procedure increased the composition of the essential oil but decreased the composition of the hydrosol; from the standpoint of main component content, the ultrasound–microwave procedure could increase the content of the main components as opposed to immersion, possibly because of the relatively high boiling points of these components, making them less prone to volatilization and dissipation.

### 2.5. PCA Analysis of Volatile Components of CAVA Hydrosols and CADB Hydrosols

**Figure 4:**
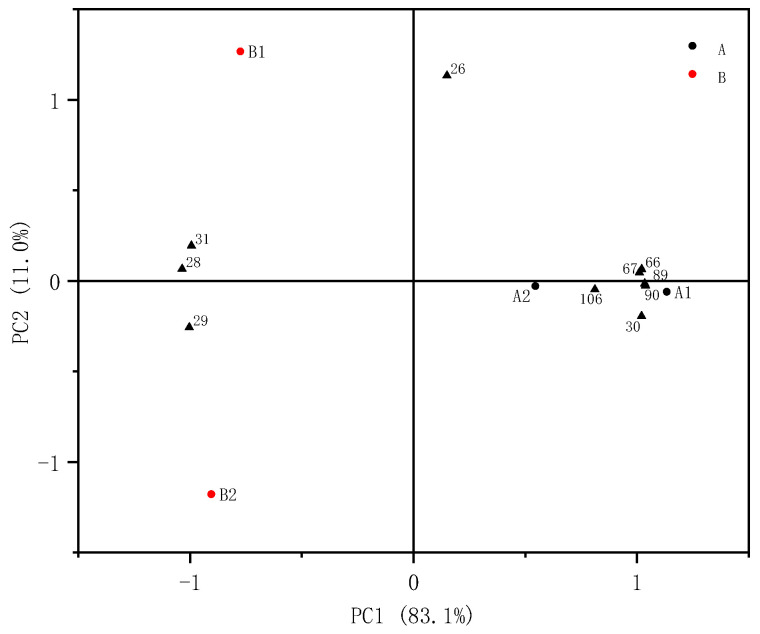
PCA analysis of main volatile components of hydrosols, biplot. A1-CAVA IH; A2-CAVA UH; B1-CADB IH; B2-CADB UH. The number 26 and other numbers indicate the main volatile components of the hydrosols (the triangles show the location of the components); specific names are shown in Table 6 (compounds).

To visually study the effects of two factors, variety and pretreatment process, on the differences in volatile components of CAVA hydrosols and CADB hydrosols, its 10 main volatile components (with a relative content > 1%) were selected for principal component (PCA) analysis, and the biplot is shown in (Figure 4). As can be seen from the figure, the contribution rates of PC1 and PC2 were 83.1% and 11.0%, respectively, with a total sum of 94.1%, which could represent the vast majority of information in the dataset of volatile constituents of CAVA hydrosols and CADB hydrosols, meeting the standard requirement of a cumulative contribution rate of 85% or higher.

The distances between A1 and B1 and A2 and B2 were greater, suggesting significant differences in the relative contents of the main components of CAVA hydrosols and CADB hydrosols under similar pretreatments. This could be attributed to disparities in the volatile component contents inherent to the varieties themselves. B1 and B2 exhibited considerable separation, while A1 and A2 were nearby, indicating that when the varieties were the same, the pretreatments had a more pronounced impact on the main volatile components of the CADB hydrosols. Conversely, the influence on the main volatile components of the CAVA hydrosols was minimal, possibly due to the large differences in the constituent types of CAVAs and CADBs, as well as the intricate synergistic, masking, and additive effects that existed among the components [27]. In PC1, the component with the highest positive loadings was geranyl acetate, and the component with the highest negative loadings was linalool; in PC2, the component with the highest positive loadings was trans-geraniol, and the component with the highest negative loadings was α-terpineol. The above results indicate that geranyl acetate, linalool, trans-geraniol, and α-terpineol were the main volatile components demonstrating the most notable variations in the contents of different hydrosols.

### 2.6. OVA Analysis of Volatile Components of CAVA Hydrosols and CADB Hydrosols

It has been documented that linalool is a natural and non-toxic compound, possessing a floral (reminiscent of lily of the valley, rose, lilac), citrus, and woody aroma [28]. The beneficial effects of linalool are inflammatory, antioxidant, antibacterial, anti-anxiety, antidepressant, etc. It is mainly used in the manufacture of pharmaceuticals, cosmetics, spices, pesticides, etc. [29] Additionally, it is utilized as a spice and flavoring agent in beverages and various food products, as well as an intermediate substance in the synthesis of vitamin E. Consequently, over 1000 tons of linalool are used globally every year, indicating its substantial value [30]. α-Terpineol has a lilac floral, citrus, and woody aroma [31] and is widely used as a flavoring component in food, tobacco, perfume, cosmetics, and cleaning products. It holds economic significance in pharmaceutical products due to its manifold beneficial effects encompassing antioxidant, anti-inflammatory, antibacterial, analgesic, antidiarrheal, anticonvulsant, anticancer, and antihypertensive properties [32]. Spathulenol exudes an herbal aroma [33], offering antioxidant, anti-inflammatory, antiproliferative, and antimycobacterial activities [34]. Geranyl acetate emits a scent reminiscent of rose and lavender [35] and exhibits promising anti-bacterial and anti-fungal characteristics with commendable thermal stability, suitable for integration into standard polymer processing methods like cellulose acetate, commonly used in the manufacture of food packaging and biomedical equipment [36]. D-limonene has a citrus lemon aroma [37], and offers various health benefits such as antioxidant, anti-diabetic, anti-cancer, anti-inflammatory, cardioprotective, gastroprotective, hepatoprotective, and immunomodulatory properties, among others. It is abundant in lemon, orange, and other citrus plants and is usually used as a fragrance and flavor additive in perfumes, soaps, and foods [38]. Nerol has a floral, grassy aroma [39] and is extensively applied in cosmetics, household detergents, and cleaners. In the food industry, it serves as a flavoring agent in foods like chewing gum and aids in food preservation due to its potent antifungal properties [40]. β-Myrcene, found in plants like lemongrass and rosemary, boasts a peppery, pungent, and floral profile [41,42]. It possesses analgesic, anti-inflammatory, anti-bacterial, and antioxidant effects, among other pharmacological effects [43]. Neryl acetate possesses a rosy, honey-sweet flavor [44], along with antibacterial, anti-inflammatory, sedative, etc., effects. It finds extensive use in the food, agricultural, and cosmetic fields and has been recognized and approved by the U.S. Food and Drug Administration as a safe food flavoring [45]. In addition, the European Food Safety Authority has assessed it as a safe feed flavoring product for use in all kinds of animals [46]. Caryophyllene oxide has a pungent, woody, clove, and slightly sweet flavor [47] and is commonly utilized as a preservative in foods, pharmaceuticals, and cosmetics, e.g., to improve oral diseases caused by oral candida [48]. Trans-geraniol features a rosy, lemony aroma [49]. Geraniol, predominantly present in essential oils like rose, ginger, lemon, etc., has neuroprotective, anti-inflammatory, and antidepressant activities, making it a popular choice in the fragrance and cosmetic sectors [50].

It is clear from the preceding that the main volatile components in the CAVA hydrosols were linalool, α-terpineol, spathulenol, geranyl acetate, trans-geraniol, d-limonene, nerol, β-myrcene, neryl acetate, and caryophyllene oxide. In the CADB hydrosols, the main volatile components were linalool, α-terpineol, trans-geraniol, nerol, and spathulenol. Most of these components are citrusy, floral, and woody, and could potentially be the foundational elements constituting the base of the aroma of CAVA hydrosols and CADB hydrosols.

The odor thresholds of 106 volatile components were retrieved and the OAV values were calculated to study the characteristic aroma components of CAVA hydrosols and CADB hydrosols. As shown in (Table 6), the threshold values of only 36 components were queried, and there were five components with OAV > 1, including three components with OAV > 10. The component with OAV > 1 in CAVA hydrosols was linalool (with a value of up to 7000 or more), followed by β-myrcene (with a value of up to 60 or more), trans-geraniol (with a value of up to 40 or more), d-limonene (with a value of around 3), and o-cymene (with a value of 2.125). The component with OAV > 1 in CADB hydrosols was linalool (with a value of up to 14,000 or more), followed by trans-geraniol (with a value of up to 40 or more), β-myrcene (value up to 30 or more), and d-limonene (value around 1). Therefore, the characteristic aroma components in CAVA hydrosols were identified as linalool, β-myrcene, trans-geraniol, d-limonene, and o-cymene, while in CADB hydrosols they were linalool, trans-geraniol, β-myrcene and d-limonene.

When the pretreatments were the same, the characteristic aroma components in the CAVA hydrosols with higher OAV values compared to the CADB hydrosols were β-myrcene and d-limonene, while the lower ones were linalool and trans-geraniol. These characteristic aroma variances can serve as a foundation for differentiation in identification.

When the varieties were the same, the OAV values of linalool, β-myrcene, trans-geraniol, and d-limonene increased, and o-cymene was depleted in the CAVA hydrosols by the ultrasound–microwave procedure compared with the immersion procedure, whereas the OAV values of the four key aromatic components in the CADB hydrosols decreased with the ultrasound–microwave procedure contrast to with the immersion procedure. Therefore, the ultrasound–microwave procedure could stimulate the aroma of CAVA hydrosols and diminish the aroma of CADB hydrosols.

### 2.7. OPLS-DA Analysis of Volatile Components of CAVA Hydrosols and CADB Hydrosols

**Figure 5:**
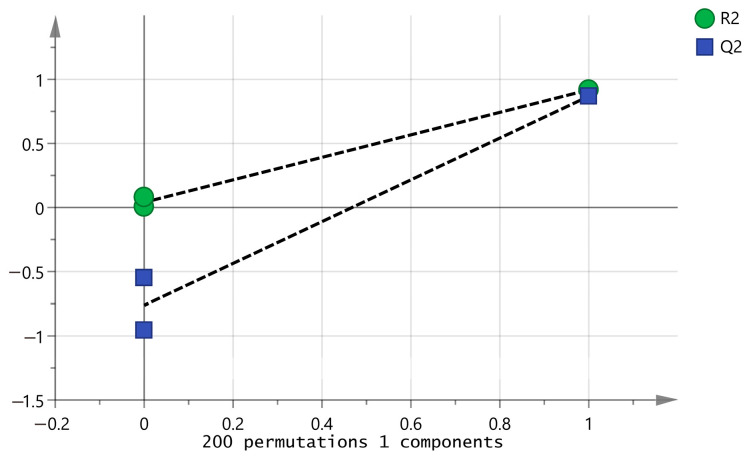
OPLS-DA model cross-validation results.

To further characterize the key aromatic components of the CAVA hydrosols and CADB hydrosols, five key aromatic components with OAV > 1 were selected for orthogonal partial least squares discriminant (OPLS-DA) analysis. The fitted parameters analyzed in this study, R^2^X = 0.998, R^2^Y = 0.918, and Q^2^ = 0.868 were assessed, with R^2^ and Q^2^ exceeding 0.5, indicating a satisfactory model fit. The cross-validation model was validated by the alignment test 200 times (Figure 5), as the intersection of the Q^2^ regression line with the y-axis was below zero, which indicates that the model is not overfitted and the model is valid, so this result can be used in distinguishing between different CAVA hydrosols and CADB hydrosols.

**Figure 6:**
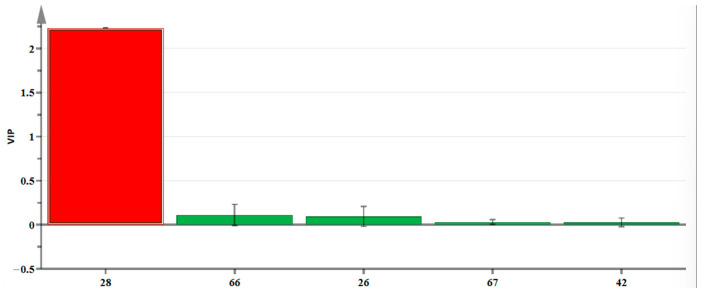
VIP diagram of OPLS-DA model. The number 26 and other numbers indicate the main volatile components of the hydrosols, with specific names shown in Table 6 (compounds); red indicates that the VIP value > 1, green indicates that the VIP value < 1.

It is typically accepted that variables with variable importance in the projection (VIP) value >1 are significantly influential to the categorization process, and the higher the value, the greater the variable’s impact on the categorization. Based on the OAV > 1 and VIP > 1 (Figure 6), one characteristic aroma component was identified, namely linalool (present in lily of the valley, rose and lilac, citrus, and woody aromas). This finding is consistent with previous research indicating linalool as the main component in the hydrosol of lime flowers [19]. Therefore, the aroma profiles of CAVA hydrosols and CADB hydrosols primarily consist of citrus, floral, and woody essences.

## 3. Materials and Methods

### 3.1. Plant Materials

The experimental materials were harvested on 20 April 2022 from the *Citrus × aurantium* L. planting base of Sichuan Shugeng Agricultural Development Co., Ltd. in Sanbanqiao Village, Guanyinqiao Town, Linshui County, Guang’an City, China. They were identified by associate researcher Xianjian Zhou of the Sichuan Academy of Traditional Chinese Medicine as the dried flower buds of *Citrus × aurantium* L., a citrus plant of the Rutaceae family, and its variant *Citrus × aurantium* ‘Daidai’.

### 3.2. Chemicals and Reagents

The chemical 2-methyl-3-heptanone was purchased from Merck Chemical Technology Co., Ltd. (Shanghai, China).

### 3.3. Preparation of Samples

After drying, the CAVAs and CADBs were comminuted using a high-speed pulverizer and sifted into 80-mesh particles for further utilization.

Immersion: firstly, 15 g of pollens and 210 mL of distilled water were combined in a 500 mL round-bottomed flask, allowed to soak for 1 h, and then extracted by heating using a steam distillation apparatus.

Ultrasound–microwave: Then, 15 g of pollens and 210 mL of distilled water were placed in a 500 mL round-bottomed flask, subjected to ultrasound cleaner and a microwave oven successively for a certain time (processing conditions: ultrasound time 7 min, ultrasound power 90 w, microwave time 75 s, microwave power 280 w). Subsequently, the mixture was extracted by heating in a steam distillation apparatus.

After the above processes, the hydro-oil was separated by the density difference method. The upper layer of essential oils was discarded, and the lower layer of water samples was collected in brown bottles and placed in a (4 ± 2) °C refrigerator light preservation spare. These were hydrosol samples.

### 3.4. Determination of Volatile Components

#### 3.4.1. Headspace Solid-Phase Microextraction

First, the SPME fiber extraction head was inserted into the gas chromatograph pretreatment for 30 min for aging at a temperature of 250 °C; then, 5.0 mL of hydrosol was added to the headspace vial, along with 10 μL of 2-methyl-3-heptanone (400 ppm) as an internal standard. The mixture was then equilibrated at 45 °C for 20 min. Subsequently, the aged head was placed in the headspace vial top headspace for adsorption at 55 °C adsorption for 30 min, followed by direct removal of the extraction head into the gas chromatography-mass spectrometry system.

#### 3.4.2. GC-MS Analysis

GC conditions: The GC oven temperature protocol included a 2 min hold at 50 °C, followed by a ramp to 120 °C at 4 °C/min and a 3 min hold (CADBs); followed by a ramp to 160 °C at 4 °C/min and a 4 min hold (CAVAs); then finally a ramp to 280 °C at 8 °C/min with a 5 min hold. The inlet temperature was set at 250 °C, using nitrogen as the carrier gas at a flow rate of 1 mL/min with a shunt ratio of 15:1.

MS conditions: The interface temperature was maintained at 280 °C, utilizing an electron bombardment (EI) source at an electron energy of 70 eV. The scanning mass range was set from 33.00 to 55.00 amu, with an electron multiplier voltage of 1000 V and a solvent delay time of 3 min.

Qualitative analysis: the volatile components of CAVA hydrosols and CADB hydrosols were analyzed by HS-SPME-GC–MS to obtain the total ion-flow chromatograms, and the components corresponding to the peaks were characterized by combining with the standard mass spectral libraries, such as NIST 107.LIB, and the pertinent literature.

Quantitative analysis: the relative content of each component was determined through the area normalization technique; the absolute content of each component was determined using the internal standard method, utilizing the concentration of 2-methyl-3-heptanone as the internal standard following the specified Formula (1):

C = (C_2_ × V_2_ × A_1_)/(A_2_ × M × 1000) (1)

where C represents the absolute content of an individual component, in mg/kg; A_1_ denotes the peak area of a single component; A_2_ indicates the peak area of an internal standard; C_2_ specifies the mass concentration of an internal standard, in μg/mL; V_2_ represents the volume of an internal standard, in μL; and M stands for the mass of the sample, in g.

OAV analysis: The content of each component obtained after quantitative analysis cannot be used as the basis for determining the key aromatic components, which can be further screened by using the volatile component OAV, and compounds with an OAV value ≥ 1 are usually regarded as contributing to the aroma characteristics, and when the OAV value is > 10, their overall aroma contribution is great [21]. After calculating the absolute content of each volatile component, the odor thresholds were determined by searching the relevant literature [51,52,53], and the odor activity value of each volatile component was calculated according to Formula (2):

OAV = C_i_/T_i_ (2)

where OAV denotes the odor activity value of a component; C_i_ represents the absolute content of a component, measured in μg/kg; and T_i_ signifies the odor threshold of a component, also in μg/kg.

### 3.5. Data Analysis

Screening searches for chemical constituents and data processing were carried out using databases like NIST 107.LIB and Microsoft Excel 2019 software; Venn plots were generated using an online website: “https://bioinfogp.cnb.csic.es/tools/venny/index.html” (accessed on 1 July 2024); cluster plotting was conducted utilizing bioinformatics tools; histograms and PCA analysis were performed using Origin 2018 software; OPLS-DA analysis was conducted using SIMCA 14.1 software. The component percentages were calculated as average values from duplicate GC-MS analyses of all the extracts.

## 4. Conclusions

In this study, CAVAs and CADBs were subjected to two pretreatments, namely immersion and ultrasound–microwave procedures, and were extracted by steam distillation to collect the hydrosols. The volatile components present in the hydrosol were concentrated and identified using HS-SPME-GC-MS. The results can provide a theoretical basis for the direction of mining the functions of the aroma components of CAVA hydrosols and CADB hydrosols. The results show that 67, 54, 32, and 26 kinds were detected in the four hydrosols, respectively, and a total of 106 kinds were detected by removing replicates. A comparison with previous studies (20~60 kinds) showed a significant increase in the number of compounds detected in lime flowers, with the main volatile components in all four hydrosols being alcohols, alkenes, and esters, with cumulative relative contents of 77.17%, 80.82%, 86.12% and 85.05%, respectively, and the high content components of both CAVA hydrosols and CADB hydrosols were identified as linalool (36.79~56.54%), α-terpineol (9.42~13.04%), and trans-geraniol (6.52~7.92%).

In terms of variety, the total components (67, 54 kinds) and unique components (66 kinds) of CAVA hydrosols far exceeded those of CADB hydrosols (32, 26 kinds)/18 kinds, respectively; the relative contents of 13 components such as d-carvone, β-myrcene, d-limonene, neryl acetate, etc., of CAVA hydrosols are greater than those of CADB hydrosols, ranging from 1 to 15-fold, with neryl acetate and geranyl acetate having the highest multiples of up to 9.85 and 15-fold; CAVA hydrosols and CADB hydrosols possess citrus, floral, and woody scents, with the distinctive aroma of CAVA hydrosols, i.e., specifically the OAV values of β-myrcene and d-limonene, surpassing those of CADB hydrosols. The characteristic aroma distinctions between the two can serve as a basis for differentiation in identification.

From the pretreatment, more volatile components were retained in the immersion (67, 32 kinds) compared to in the ultrasound–microwave procedure (54, 26 kinds). The relative contents of the main components, i.e., linalool (44.38%) and α-terpineol (10.81%, 13.04%), were higher in the ultrasound–microwave procedure than in the immersion (36.79%; 9.42%, 11.99%, respectively); the ultrasound–microwave procedure was favorable for the stimulation of the aroma of CAVA hydrosols (increased OAV of linalool, β-myrcene, trans-geraniol and d-limonene), but it would diminish the aroma of the CADB hydrosols (decreased OAV of linalool, trans-geraniol, β-myrcene and d-limonene).

CADB hydrosols are suitable for use only in the daily chemical industry, while in addition to the pharmaceutical and daily chemical industry, CAVA hydrosols may be used in the food industry based on edible components like d-limonene, neryl acetate, geranyl acetate, caryophyllene oxide, etc., which have a wide range of benefits. These hydrosols can be collected through an appropriate process as per specific needs for utilization. Importantly, the safety profile of using these hydrosols in food is superior to pharmaceuticals, with long-term consumption potentially yielding preventive health benefits that drugs may not provide. Additionally, CAVA hydrosols have a unique food property and can be directly used in food development; their application prospects are broad. This study provides a research basis for the in-depth exploration of the basis of the medicine food homology properties of CAVAs as well as the in-depth development and application of the CAVA hydrosols and CADB hydrosols and could potentially enhance the efficiency of waste resource utilization and drive advancements in relevant industries.

## Figures and Tables

**Table 1:** Analysis of the volatile components of CAVA IH.

No.	Retention Time (min)	Compound	Molecular Formula	Compound Type	Absolute Content (mg/kg)	Relative Content (%)
1	6.434	3-Heptanone,4-methyl	C_8_H_16_O	Ketone	0.0005	0.01
2	6.785	2-Heptanone,3-methyl-	C_8_H_16_O	Ketone	0.0029	0.07
3	7.103	3-Phenyl-2-butanol	C_10_H_14_O	Alcohol	0.0009	0.02
4	7.353	Benzene,1-ethyl-2-methyl	C_9_H_12_	Aromatic hydrocarbon	0.0009	0.02
5	7.505	Benzene,1,2,3-trimethyl-	C_9_H_12_	Aromatic hydrocarbon	0.0143	0.33
6	7.615	Nonane,5-methylene-	C_10_H_20_	Alkene	0.0039	0.09
7	7.725	Allyl butyrate	C_7_H_12_O_2_	Ester	0.0039	0.09
8	7.806	Diallyl carbonate	C_7_H_10_O_3_	Ester	0.0003	0.01
9	7.865	2-Norpinene-2-ethanol,6,6-dimethyl-	C_11_H_18_O	Alcohol	0.0022	0.05
10	8.256	β-Myrcene	C_10_H_16_	Alkene	0.0769	1.80
11	8.656	α-Phellandrene	C_10_H_16_	Alkene	0.0117	0.28
12	8.749	trans, trans-2,8-Decadiene	C_10_H_18_	Alkene	0.0059	0.14
13	9.064	α-Terpinene	C_10_H_16_	Alkene	0.0130	0.31
14	9.232	p-Cymene	C_10_H_14_	Aromatic hydrocarbon	0.0024	0.06
15	9.354	o-Cymene	C_10_H_14_	Aromatic hydrocarbon	0.0085	0.20
16	9.455	D-Limonene	C_10_H_16_	Alkene	0.0937	2.20
17	9.827	trans-β-Ocimene	C_10_H_16_	Alkene	0.0221	0.52
18	10.174	cis-β-Ocimene	C_10_H_16_	Alkene	0.0316	0.74
19	10.491	γ-Terpinene	C_10_H_16_	Alkene	0.0085	0.20
20	10.972	α-Methyl-α-[4-methyl-3-pentenyl] oriranemethanol	C_10_H_18_O_2_	Alcohol	0.0125	0.29
21	11.513	2-Carene	C_10_H_16_	Alkene	0.0275	0.64
22	12.166	Linalool	C_10_H_18_O	Alcohol	1.5674	36.79
23	13.006	1,3,8-p-Menthatriene	C_10_H_14_	Alkene	0.0235	0.55
24	13.430	Neo-alloocimene, stab.	C_10_H_16_	Alkene	0.0091	0.21
25	14.624	Terpinen-4-ol	C_10_H_18_O	Alcohol	0.0073	0.17
26	15.149	α-Terpineol	C_10_H_18_O	Alcohol	0.4013	9.42
27	16.126	Octahydro-5-(2-octyldecyl)-4,7-methano-1H-indene	C_28_H_52_	Indene	0.0004	0.01
28	16.451	Nerol	C_10_H_18_O	Alcohol	0.0841	1.97
29	17.061	D-Carvone	C_10_H_14_O	Ketone	0.0071	0.17
30	17.366	trans-Geraniol	C_10_H_18_O	Alcohol	0.3119	7.32
31	17.903	Butanenitrile	C_4_H_7_N	Other	0.0020	0.05
32	18.085	4,6-Dimethyldodecane	C_14_H_30_	Alkane	0.0019	0.04
33	18.272	Isoborneol	C_10_H_18_O	Alcohol	0.0018	0.04
34	18.561	Ethylene glycol diallyl ether	C_8_H_14_O_2_	Ether	0.0006	0.01
35	18.668	Butanal,4-[(tetrahydro-2H-pyran-2-yl)oxy]-	C_9_H_16_O_3_	Aldehyde	0.0013	0.03
36	19.035	2,3,5,8-Tetramethyldecane	C_14_H_30_	Alkane	0.0004	0.01
37	19.478	Cadala-1(10),3,8-triene	C_15_H_22_	Alkene	0.0007	0.02
38	19.619	2,6,11-Trimethyldodecane	C_15_H_32_	Alkane	0.0008	0.02
39	20.465	α-Terpinyl acetate	C_12_H_20_O_2_	Ester	0.0047	0.11
40	21.093	Neryl acetate	C_12_H_20_O_2_	Ester	0.0647	1.52
41	21.903	Geranyl acetate	C_12_H_20_O_2_	Ester	0.1066	2.50
42	22.528	Tetradecane	C_14_H_30_	Alkane	0.0006	0.01
43	24.016	Artemesia alcohol	C_10_H_18_O	Alcohol	0.0022	0.05
44	24.478	7-Tetracyclo[6.2.1.0(3.8)0(3.9)]undecanol, 4,4,11,11-tetramethyl-	C_15_H_24_O	Alcohol	0.0100	0.24
45	24.677	(Z)-β-farnesene	C_15_H_24_	Alkene	0.0035	0.08
46	24.802	4,5-dimethyl-Biphenylene,1,2,3,6,7,8,8a,8b-octahydro-4,5-dimethyl-	C_14_H_20_	Aromatic hydrocarbon	0.0045	0.11
47	25.221	Dehydro aromadendrene	C_15_H_22_	Alkene	0.0039	0.09
48	25.714	1-Heptatriacotanol	C_37_H_76_O	Alcohol	0.0029	0.07
49	25.849	α-Muurolene	C_15_H_24_	Alkene	0.0027	0.06
50	26.210	γ-Muurolene	C_15_H_24_	Alkene	0.0045	0.11
51	26.304	Phenol,2,4-bis(1,1-dimethylethyl)-	C_14_H_22_O	Phenolic	0.0049	0.12
52	26.437	Calamenene	C_15_H_22_	Alkene	0.0064	0.15
53	26.671	8,9-dehydro-Neoisolongifolene	C_15_H_22_	Alkene	0.0033	0.08
54	26.917	2-Chlorobenzoic acid, dodec-9-ynyl ester	C_19_H_25_ClO_2_	Ester	0.0066	0.15
55	27.417	trans-Nerolidol	C_15_H_26_O	Alcohol	0.0319	0.75
56	27.682	Spathulenol	C_15_H_24_O	Alcohol	0.1937	4.55
57	27.773	Caryophyllene oxide	C_15_H_24_O	Other	0.0426	1.00
58	27.960	(+)-Viridiflorol	C_15_H_26_O	Alcohol	0.0084	0.20
59	28.069	2,2,4-Trimethyl-1,3-pentanediol diisobutyrate	C_16_H_30_O_4_	Ester	0.0081	0.19
60	28.195	Ledol	C_15_H_26_O	Alcohol	0.0097	0.23
61	29.183	α-Cadinol	C_15_H_26_O	Alcohol	0.0249	0.58
62	29.481	Tricyclo[5.2.2.0(1,6)]undecan-3-ol,2-methylene-6,8,8-trimethyl	C_15_H_24_O	Alcohol	0.0136	0.32
63	29.772	7R,8R-8-Hydroxy-4-isopropylidene-7-methylbicyclo[5.3.1]undec-1-ene	C_15_H_24_O	Alkene	0.0090	0.21
64	30.067	10-Methylnonadecane	C_20_H_42_	Alkane	0.0021	0.05
65	30.172	5-Butylnonane	C_13_H_28_	Alkane	0.0011	0.03
66	30.336	6-Isopropenyl-4,8a-dimethyl-1,2,3,5,6,7,8,8a-octahydro-naphthalen-2-ol	C_15_H_24_O	Alcohol	0.0024	0.06
67	31.239	Azulene,1,4-dimethyl-7-(1-methylethyl)-	C_15_H_18_	Alkane	0.0130	0.31

**Table 2:** Analysis of the volatile components of CAVA UH.

No.	Retention Time (min)	Compound	Molecular Formula	Compound Type	Absolute Content (mg/kg)	Relative Content (%)
1	6.761	2-Heptanone,3-methyl-	C_8_H_16_O	Ketone	0.0027	0.06
2	7.091	Bicyclo[3.1.0]hexane-6,6-dicarbonitrile	C_8_H_8_N_2_	Other	0.0011	0.02
3	7.493	Benzene,1,2,3-trimethyl-	C_9_H_12_	Aromatic hydrocarbon	0.0123	0.26
4	7.603	2-Methyl-1-hexene	C_7_H_14_	Alkene	0.0030	0.06
5	7.710	Allyl butyrate	C_7_H_12_O_2_	Ester	0.0024	0.05
6	8.243	β-Myrcene	C_10_H_16_	Alkene	0.0810	1.73
7	8.644	α-Phellandrene	C_10_H_16_	Alkene	0.0093	0.20
8	8.737	cis-7-Dodecen-1-ol	C_12_H_24_O	Alcohol	0.0046	0.10
9	9.053	α-Terpinene	C_10_H_16_	Alkene	0.0111	0.24
10	9.220	m-Cymene	C_10_H_14_	Aromatic hydrocarbon	0.0026	0.06
11	9.447	D-Limonene	C_10_H_16_	Alkene	0.1025	2.19
12	9.819	trans-β-Ocimene	C_10_H_16_	Alkene	0.0243	0.52
13	10.167	cis-β-Ocimene	C_10_H_16_	Alkene	0.0331	0.71
14	10.483	γ-Terpinene	C_10_H_16_	Alkene	0.0106	0.23
15	10.964	α-Methyl-α-[4-methyl-3-pentenyl] oriranemethanol	C_10_H_18_O_2_	Alcohol	0.0178	0.38
16	11.510	2-Carene	C_10_H_16_	Alkene	0.0340	0.73
17	12.162	Linalool	C_10_H_18_O	Alcohol	2.0741	44.38
18	13.008	1,3,8-p-Menthatriene	C_10_H_14_	Alkene	0.0187	0.40
19	13.426	Neo-alloocimene, stab.	C_10_H_16_	Alkene	0.0070	0.15
20	14.619	Terpinen-4-ol	C_10_H_18_O	Alcohol	0.0101	0.22
21	15.145	α-Terpineol	C_10_H_18_O	Alcohol	0.5052	10.81
22	16.136	Levoverbenone	C_10_H_14_O	Ketone	0.0021	0.04
23	16.450	Nerol	C_10_H_18_O	Alcohol	0.0914	1.96
24	16.969	D-Carvone	C_10_H_14_O	Ketone	0.0132	0.28
25	17.353	trans-Geraniol	C_10_H_18_O	Alcohol	0.3413	7.30
26	17.986	(E)-citral	C_10_H_16_O	Aldehyde	0.0013	0.03
27	18.055	4-Methylundecane	C_12_H_26_	Alkane	0.0020	0.04
28	18.282	Citronellal	C_10_H_18_O	Aldehyde	0.0022	0.05
29	18.666	Isohexylpentyl sulfite	C_11_H_24_O_3_S	Ester	0.0010	0.02
30	19.050	Oxalic acid, allyl nonyl ester	C_14_H_24_O_4_	Ester	0.0008	0.02
31	20.463	α-Terpinyl acetate	C_12_H_20_O_2_	Ester	0.0047	0.10
32	21.099	Neryl acetate	C_12_H_20_O_2_	Ester	0.0597	1.28
33	21.914	Geranyl acetate	C_12_H_20_O_2_	Ester	0.0909	1.94
34	22.540	Tetradecane	C_14_H_30_	Alkane	0.0006	0.01
35	24.029	Isovaleric anhydride	C_10_H_18_O_3_	Acid	0.0013	0.03
36	24.478	7-Tetracyclo[6.2.1.0(3.8)0(3.9)]undecanol, 4,4,11,11-tetramethyl-	C_15_H_24_O	Alcohol	0.0096	0.21
37	24.686	trans-Caryophyllene	C_15_H_24_	Alkene	0.0011	0.02
38	24.798	4,5-dehydro-Isolongifolene	C_15_H_22_	Alkene	0.0020	0.04
39	25.541	Dotriacontane	C_32_H_66_	Alkane	0.0021	0.04
40	25.731	n-Eicosane	C_20_H_42_	Alkane	0.0023	0.05
41	26.308	Phenol,2,4-bis(1,1-dimethylethyl)-	C_14_H_22_O	Phenolic	0.0079	0.17
42	26.676	8,9-dehydro-Neoisolongifolene	C_15_H_22_	Alkene	0.0017	0.04
43	26.920	4,6-Cholestadiene-3-one, 2,4-dinitrophenylhydrazone	C_33_H_46_N_4_O_4_	Phenylhydrazone	0.0031	0.07
44	27.097	Caryophyllene oxide	C_15_H_24_O	Other	0.0020	0.04
45	27.458	trans-Nerolidol	C_15_H_26_O	Alcohol	0.0076	0.16
46	27.669	Spathulenol	C_15_H_24_O	Alcohol	0.1626	3.48
47	28.070	2,2,4-Trimethyl-1,3-pentanediol diisobutyrate	C_16_H_30_O_4_	Ester	0.0067	0.14
48	28.881	(−)-Spathulenol	C_15_H_24_O	Alcohol	0.0283	0.61
49	29.188	α-Cadinol	C_15_H_26_O	Alcohol	0.0119	0.26
50	29.546	3,5-Dehydro-6-methoxy-trimethylacetate-cholest-22-en-21-ol	C_33_H_54_O_3_	Acid	0.0075	0.16
51	29.869	Oxalic acid, 6-ethyloct-3-yl propyl ester	C_15_H_28_O_4_	Ester	0.0050	0.11
52	30.748	Tetracosane	C_24_H_50_	Alkane	0.0019	0.04
53	31.263	Guaiazulene	C_15_H_18_	Alkane	0.0043	0.09
54	31.579	Oxalic acid, allyl hexadecyl ester	C_21_H_38_O_4_	Ester	0.0016	0.03

**Table 3:** Analysis of the volatile components of CADB IH.

No.	Retention Time (min)	Compound	Molecular Formula	Compound Type	Absolute Content (mg/kg)	Relative Content (%)
1	11.223	2-Heptanone,3-methyl-	C_8_H_16_O	Ketone	0.0015	0.02
2	12.883	β-Myrcene	C_10_H_16_	Alkene	0.0570	0.93
3	13.259	α-Phellandrene	C_10_H_16_	Alkene	0.0065	0.11
4	13.689	α-Terpinene	C_10_H_16_	Alkene	0.0047	0.08
5	13.922	Neopentyl dihydrocinnamate	C_14_H_20_O_2_	Ester	0.0012	0.02
6	13.987	Benzene,1-ethyl-3,5-dimethyl-	C_10_H_14_	Aromatic hydrocarbon	0.0017	0.03
7	14.079	D-Limonene	C_10_H_16_	Alkene	0.0464	0.76
8	14.531	trans-β-Ocimene	C_10_H_16_	Alkene	0.0125	0.20
9	14.863	cis-β-Ocimene	C_10_H_16_	Alkene	0.0185	0.30
10	15.136	γ-Terpinene	C_10_H_16_	Alkene	0.0042	0.07
11	15.605	α-Methyl-α-[4-methyl-3-pentenyl] oriranemethanol	C_10_H_18_O_2_	Alcohol	0.0360	0.59
12	16.806	Linalool	C_10_H_18_O	Alcohol	3.4712	56.54
13	17.272	(−)-Carveol	C_10_H_16_O	Alcohol	0.0024	0.04
14	17.485	Cosmene	C_10_H_14_	Alkene	0.0088	0.14
15	17.720	(−)-trans-Pinocarveol	C_10_H_16_O	Alcohol	0.0054	0.09
16	17.859	1-Methylene-2-methyl-3-isopropenylcyclopentane	C_10_H_16_	Cycloalkane	0.0043	0.07
17	18.430	Pinocarvone	C_10_H_14_O	Ketone	0.0023	0.04
18	18.971	(−)-Terpinen-4-ol	C_10_H_16_	Alcohol	0.0193	0.31
19	19.483	α-Terpineol	C_10_H_18_O	Alcohol	0.7361	11.99
20	20.379	cis-Carveol	C_10_H_16_O	Alcohol	0.0127	0.21
21	20.534	Nerol	C_10_H_18_O	Alcohol	0.2149	3.50
22	20.944	(−)-Carvone	C_10_H_14_O	Ketone	0.0036	0.06
23	21.192	trans-Geraniol	C_10_H_18_O	Alcohol	0.4865	7.92
24	21.789	Isoborneol	C_10_H_18_O	Alcohol	0.0012	0.02
25	22.000	Sulfurous acid,isohexyl pentyl ester	C_11_H_24_O_3_S	Ester	0.0009	0.01
26	23.381	Neryl acetate	C_12_H_20_O_2_	Ester	0.0123	0.20
27	23.771	Geranyl acetate	C_12_H_20_O_2_	Ester	0.0134	0.22
28	24.718	Dehydro aromadendrene	C_15_H_22_	Alkene	0.0047	0.08
29	25.627	Phenol,2,4-bis(1,1-dimethylethyl)-	C_14_H_22_O	Phenolic	0.0083	0.13
30	25.884	7,9-Dimethylhexadecane	C_18_H_38_	Alkane	0.0013	0.02
31	26.492	Spathulenol	C_15_H_24_O	Alcohol	0.1041	1.70
32	27.285	(−)-Spathulenol	C_15_H_24_O	Alcohol	0.0054	0.09

**Table 4:** Analysis of the volatile components of CADB UH.

No.	Retention Time (min)	Compound	Molecular Formula	Compound Type	Absolute Content (mg/kg)	Relative Content (%)
1	11.282	3,4-Dimethyl-2-hexanone	C_8_H_16_O	Ketone	0.0097	0.18
2	12.885	β-Myrcene	C_10_H_16_	Alkene	0.0435	0.79
3	14.081	D-Limonene	C_10_H_16_	Alkene	0.0310	0.56
4	14.537	trans-β-Ocimene	C_10_H_16_	Alkene	0.0072	0.13
5	14.874	cis-β-Ocimene	C_10_H_16_	Alkene	0.0115	0.21
6	15.608	cis-Linalool oxide (furanoid)	C_10_H_18_O_2_	Alcohol	0.0399	0.72
7	16.081	3,4-Dimethylbenzyl alcohol	C_9_H_12_O	Alcohol	0.0028	0.05
8	16.159	trans-Linalool oxide (furans)	C_10_H_18_O_2_	Alcohol	0.0165	0.30
9	16.788	Linalool	C_10_H_18_O	Alcohol	3.1096	56.44
10	17.490	Cosmene	C_10_H_14_	Alkene	0.0029	0.05
11	17.716	(−)-trans-Pinocarveol	C_10_H_16_O	Alcohol	0.0038	0.07
12	18.979	(−)-Terpinen-4-ol	C_10_H_16_	Alcohol	0.0169	0.31
13	19.493	α-Terpineol	C_10_H_18_O	Alcohol	0.7186	13.04
14	20.370	cis-Carveol	C_10_H_16_O	Alcohol	0.0043	0.08
15	20.536	Nerol	C_10_H_18_O	Alcohol	0.1795	3.26
16	20.931	D-Carvone	C_10_H_14_O	Ketone	0.0024	0.04
17	21.190	trans-Geraniol	C_10_H_18_O	Alcohol	0.3591	6.52
18	21.643	5-Ethyl-5-methyldecane	C_13_H_28_	Alkane	0.0016	0.03
19	21.795	Glutaric acid, tridec-2-yn-1-yl 2-decyl ester	C_28_H_50_O_4_	Ester	0.0017	0.03
20	23.379	Neryl acetate	C_12_H_20_O_2_	Ester	0.0069	0.13
21	23.774	Geranyl acetate	C_12_H_20_O_2_	Ester	0.0074	0.13
22	24.718	4a,5-Dimethyl-3-(prop-1-en-2-yl)-1,2,3,4,4a,5,6,7-octahydronaphthalen-1-ol	C_15_H_24_O	Alcohol	0.0021	0.04
23	24.764	(+)-Cycloheterophyllin-5-ol	C_15_H_24_O	Alcohol	0.0025	0.05
24	25.627	2,4-Bis(1,1-dimethylethyl) phenol	C_14_H_22_O	Phenolic	0.0065	0.12
25	26.491	Spathulenol	C_15_H_24_O	Alcohol	0.1147	2.08
26	27.281	(−)-Spathulenol	C_15_H_24_O	Alcohol	0.0034	0.06

**Table 5:** Analysis of the unique and shared volatile components of the 4 hydrosols.

	Compound Categories	CAVA IH	CAVA UH	CADB IH	CADB UH
Specific compounds	Alcohols	8	1	1	4
	Alkenes	8	3	-	-
	Esters	2	3	1	1
	Alkanes	5	4	1	1
	Aromatic hydrocarbons	4	1	1	-
	Ketones	1	1	2	1
	Aldehydes	1	2	-	-
	Ether	1	-	-	-
	Indene	1	-	-	-
	Acids	-	2	-	-
	Phenylhydrazone	-	1	-	-
	Cycloalkane	-	-	1	-
	Others	1	1	-	-
Total	-	32	19	7	7
Shared compounds	Alkenes	4			
	Alcohols	6			
	Esters	2			
	Phenolic	1			
Total	-	13			

**Table 6:** OAV analysis of volatile components of hydrosols.

No.	Compounds	Odor Descriptors	Odor Threshold (mg/kg)	OAV			
				CAVA IH	CAVA UH	CADB IH	CADB UH
Ketone							
1	3-Heptanone,4-methyl ^#^ (1)		-	-	-	-	-
2	(−)-Carvone ^#^ (2)	Grass, menthol	0.007	-	-	0.514	-
3	3,4-Dimethyl-2-hexanone ^#^ (3)		-	-	-	-	-
4	2-Heptanone,3-methyl- (4)		-	-	-	-	-
5	D-Carvone (5)	Floral, lingering orchid	0.16	0.044	0.083	-	0.015
6	Pinocarvone ^#^ (6)	Lingering orchid	-	-	-	-	-
7	Levoverbenone ^#^ (7)	Strong minty, camphoraceous	-	-	-	-	-
Alcohol							
1	3-Phenyl-2-butanol ^#^ (8)		-	-	-	-	-
2	2-Norpinene-2-ethanol,6,6-dimethyl- ^#^ (9)		-	-	-	-	-
3	Artemesia alcohol ^#^ (10)		-	-	-	-	-
4	1-Heptatriacotanol ^#^ (11)	Grease fragrance	-	-	-	-	-
5	(+)-Viridiflorol ^#^ (12)	Peppery, spicy, camphoraceous	-	-	-	-	-
6	Ledol ^#^ (13)	Tea, fruit sweetness	-	-	-	-	-
7	Tricyclo[5.2.2.0(1,6)]undecan-3-ol,2-methylene-6,8,8-trimethyl ^#^ (14)		-	-	-	-	-
8	6-Isopropenyl-4,8a-dimethyl-1,2,3,5,6,7,8,8a-octahydro-naphthalen-2-ol ^#^ (15)		-	-	-	-	-
9	cis-7-Dodecen-1-ol ^#^ (16)		-	-	-	-	-
10	(-)-Carveol ^#^ (17)	Mint, green, herbal, coriander, spicy	0.25	-	-	0.010	-
11	(-)-trans-Pinocarveol (18)	Green, wood	-	-	-	-	-
12	cis-Carveol (19)	Citrus, spearmint	0.25	-	-	0.051	0.017
13	cis-Linalool oxide (furanoid) * (20)	Earthy, floral, sweet wood	0.32	0.039	0.056	0.113	0.125
14	3,4-Dimethylbenzyl alcohol ^#^ (21)		-	-	-	-	-
15	trans-Linalool oxide (furans) ^#^ (22)	sweet scent	0.32	-	-	-	0.052
16	4a,5-Dimethyl-3-(prop-1-en-2-yl)-1,2,3,4,4a,5,6,7-octahydronaphthalen-1-ol ^#^ (23)		-	-	-	-	-
17	(+)-Cycloisolongifol-5-ol ^#^ (24)		-	-	-	-	-
18	(−)-Terpinen-4-ol (25)	Nutmeg, musk	1.2	-	-	0.016	0.014
19	trans-Geraniol (26)	Passion fruit, lemon, rose	0.0075	41.587	45.507	64.867	47.880
20	(−)-Spathulenol (27)	Pungent, loamy, woody	-	-	-	-	-
21	Linalool * (28)	Floral (lily of the valley, rose, lilac), citrus, woody	0.00022	7124.55	9427.73	15778.182	14134.545
22	α-Terpineol * (29)	Lilac floral, citrus, woody	1.2	0.334	0.421	0.613	0.599
23	Spathulenol * (30)	Earthy, herbal, fruity	-	-	-	-	-
24	Nerol * (31)	Flowery, grassy	0.68	0.124	0.134	0.316	0.264
25	Terpinen-4-ol (32)	Wooden, fresh	1.2	0.006	0.008	-	-
26	7-Tetracyclo[6.2.1.0(3.8)0(3.9)]undecanol, 4,4,11,11-tetramethyl- (33)		-	-	-	-	-
27	trans-Nerolidol (34)	Fir, pine incense	0.25	0.128	0.030	-	-
28	α-Cadinol (35)	Herbs, herbal medicine, mullein	-	-	-	-	-
29	Isoborneol (36)	Camphoraceous	0.0085	0.212	-	0.141	-
Aromatic hydrocarbon							
1	Benzene,1-ethyl-2-methyl ^#^ (37)		-	-	-	-	-
2	p-Cymene ^#^ (38)	Subtle citrus, carrot	0.005	0.48	-	-	-
3	4,5-dimethyl-Biphenylene,1,2,3,6,7,8,8a,8b-octahydro-4,5-dimethyl- ^#^ (39)		-	-	-	-	-
4	Benzene,1-ethyl-3,5-dimethyl- ^#^ (40)		-	-	-	-	-
5	Benzene,1,2,3-trimethyl- (41)	Aromatic	-	-	-	-	-
6	o-Cymene ^#^ (42)		0.004	2.125	-	-	-
7	m-Cymene ^#^ (43)		0.8	-	0.003	-	-
Alkane							
1	4,6-Dimethyldodecane ^#^ (44)		-	-	-	-	-
2	2,3,5,8-Tetramethyldecane ^#^ (45)		-	-	-	-	-
3	2,6,11-Trimethyldodecane ^#^ (46)	Tasteless	-	-	-	-	-
4	10-Methylnonadecane ^#^ (47)		-	-	-	-	-
5	5-Butylnonane ^#^ (48)		-	-	-	-	-
6	4-Methylundecane ^#^ (49)		-	-	-	-	-
7	Dotriacontane ^#^ (50)	Tasteless	-	-	-	-	-
8	n-Eicosane ^#^ (51)	Glutinous rice	-	-	-	-	-
9	Tetracosane ^#^ (52)	Tasteless	-	-	-	-	-
10	7,9-Dimethylhexadecane ^#^ (53)		-	-	-	-	-
11	5-Ethyl-5-methyldecane ^#^ (54)		-	-	-	-	-
12	Tetradecane (55)	Waxiness	0.005	0.120	0.120	-	-
13	Guaiazulene (56)		-	-	-	-	-
Alkene							
1	Nonane,5-methylene- ^#^ (57)		-	-	-	-	-
2	(Z)-β-farnesene ^#^ (58)	Citrus, floral	0.087	0.040	-	-	-
3	α-Muurolene ^#^ (59)	Fresh flower	0.0075	0.360	-	-	-
4	γ-Muurolene ^#^ (60)	Fresh flower	-	-	-	-	-
5	7R,8R-8-Hydroxy-4-isopropylidene-7-methylbicyclo [5.3.1] undec-1-ene ^#^ (61)		-	-	-	-	-
6	2-Methyl-1-hexene ^#^ (62)		-	-	-	-	-
7	trans-Caryophyllene ^#^ (63)	Sweet, woody, peppery	0.064	-	0.017	-	-
8	4,5-dehydro-Isolongifolene ^#^ (64)		-	-	-	-	-
9	cis-β-Ocimene * (65)	Grassy, floral with orange blossom oil	0.034	0.929	0.974	0.544	0.338
10	β-Myrcene * (66)	Peppery, spicy, floral	0.0012	64.083	67.500	47.500	36.250
11	D-Limonene * (67)	Citrus, lemon	0.034	2.756	3.015	1.365	0.912
12	trans-β-Ocimene * (68)	Green, citrus, mint	0.034	0.650	0.715	0.368	0.212
13	α-Phellandrene (69)	Black pepper	0.036	0.325	0.258	0.181	-
14	α-Terpinene (70)	Woody, tea	0.08	0.163	0.139	0.059	-
15	γ-Terpinene (71)	Citrus	1	0.009	0.011	0.004	-
16	2-Carene (72)	Green grassy	0.037	0.743	0.919	-	-
17	Neo-alloocimene, stab. (73)	Grassy, floral with orange blossom oil	0.034	0.268	0.206	-	-
18	trans, trans-2,8-Decadiene ^#^ (74)		-	-	-	-	-
19	Calamenene ^#^ (75)	Vanilla, Camphoraceous, medicinal	-	-	-	-	-
20	Cosmene * (76)		-	-	-	-	-
21	8,9-dehydro-Neoisolongifolene (77)		-	-	-	-	-
22	Dehydro aromadendrene (78)		-	-	-	-	-
23	Cadala-1(10),3,8-triene ^#^ (79)		-	-	-	-	-
24	1,3,8-p-Menthatriene ^#^ (80)		-	-	-	-	-
Ester							
1	2-Chlorobenzoic acid, dodec-9-ynyl ester ^#^ (81)		-	-	-	-	-
2	Diallyl carbonate ^#^ (82)		-	-	-	-	-
3	Oxalic acid, allyl nonyl ester ^#^ (83)		-	-	-	-	-
4	Oxalic acid, 6-ethyloct-3-yl propyl ester ^#^ (84)		-	-	-	-	-
5	Oxalic acid, allyl hexadecyl ester ^#^ (85)		-	-	-	-	-
6	Neopentyl dihydrocinnamate ^#^ (86)		-	-	-	-	-
7	Glutaric acid, tridec-2-yn-1-yl 2-decyl ester ^#^ (87)		-	-	-	-	-
8	Sulfurous acid,isohexyl pentyl ester (88)		-	-	-	-	-
9	Neryl acetate * (89)	Rose fragrance, honey sweetness	2	0.032	0.030	0.006	0.003
10	Geranyl acetate * (90)	Rose, lavender	0.15	0.711	0.606	0.089	0.049
11	Allyl butyrate (91)		-	-	-	-	-
12	α-Terpinyl acetate (92)	Pine fragrance, woody fragrance	2.5	0.002	0.002	-	-
13	2,2,4-Trimethyl-1,3-pentanediol diisobutyrate (93)		-	-	-	-	-
Aldehyde							
1	Butanal,4-[(tetrahydro-2H-pyran-2-yl) oxy]- ^#^ (94)		-	-	-	-	-
2	(E)-citral ^#^ (95)		-	-	-	-	-
3	Citronellal ^#^ (96)	Fruity	0.0035	-	0.629	-	-
Phenolic							
1	2,4-Bis(1,1-dimethylethyl) pheno^l^ * (97)	Phenolic, resinous	0.5	0.010	0.016	0.017	0.013
Cycloalkane							
1	1-Methylene-2-methyl-3-isopropenylcyclopentane ^#^ (98)		-	-	-	-	-
Acid							
1	Isovaleric anhydride ^#^ (99)		-	-	-	-	-
2	3,5-Dehydro-6-methoxy-trimethylacetate-cholest-22-en-21-ol ^#^ (100)		-	-	-	-	-
Ether							
1	Ethylene glycol diallyl ether ^#^ (101)		-	-	-	-	-
Phenylhydrazone							
1	4,6-Cholestadiene-3-one, 2,4-dinitrophenylhydrazone ^#^ (102)		-	-	-	-	-
Indene							
1	Octahydro-5-(2-octyldecyl)-4,7-methano-1H-indene ^#^ (103)		-	-	-	-	-
Other							
1	Bicyclo[3.1.0]hexane-6,6-dicarbonitrile ^#^ (104)		-	-	-	-	-
2	Butanenitrile ^#^ (105)		-	-	-	-	-
3	Caryophyllene oxide (106)	Pungent, woody, clove, some sweetness	0.41	0.112	0.005	-	-

* Compounds common to the four hydrosols; ^#^ compounds specific to the four hydrosols; “-”: the compound is not detected. Numbers (1)–(106) are the compound numbers in the study.

## Data Availability

Data are contained within the article.

## References

[1] Chinese Pharmacopoeia Commission (2020). Pharmacopoeia of the People’s Republic of China.

[2] Sarrou E., Chatzopoulou P., Dimassi-Theriou K., Therios I. (2013). Volatile constituents and antioxidant activity of peel, flowers and leaf oils of *Citrus aurantium* L. growing in Greece. Molecules.

[3] Park A., Yang Y.J., Lee Y.H., Jung H.Y., Kim T.D., Noh J.Y., Lee S.J., Yoon S.R. (2022). *Aurantii Fructus Immaturus* enhances natural killer cytolytic activity and anticancer efficacy in vitro and in vivo. Front. Med..

[4] Gao T.H., Jiang M.Y., Deng B., Zhang Z., Fu Q., Fu C.M. (2021). *Aurantii Fructus*: A systematic review of ethnopharmacology, phytochemistry and pharmacology. Phytochem. Rev..

[5] Li H.H., Liu X., Huang S. (2022). Chinese *Aurantii Fructus* industry development status and Jiangjin *Aurantii Fructus* development prospect analysis. China Fruit News.

[6] National Administration of Traditional Chinese Medicine, Editorial Committee of Chinese Materia Medica (1999). Chinese Materia Medica.

[7] Chinese Academy of Sciences Editorial Committee of Flora of China (1997). Flora of China.

[8] Shen C.Y., Wang T.X., Zhang X.M., Jiang J.G. (2017). Various Antioxidant Effects Were Attributed to Different Components in the Dried Blossoms of *Citrus aurantium* L. var. amara Engl. J. Agric. Food Chem..

[9] Zhang H.X., Huang T., Liao X.N., Zhou Y.H., Chen S.X., Chen J., Xiong W.M. (2022). Extraction of camphor tree essential oil by steam distillation and supercritical CO_2_ extraction. Molecules.

[10] Li X.Y., Hao Y.F., Hao Z.X., Jiang J.G., Liu Q., Shen Q., Liu L., Yi Y.K., Shen C.Y. (2021). Inhibitory effect of chloroform extracts from *Citrus aurantium* L. var. amara Engl. on fat accumulation. Phytomedicine.

[11] Shen C.Y., Wan L., Wang T.X., Jiang J.G. (2019). *Citrus aurantium* L. var. amara Engl. inhibited lipid accumulation in 3T3-L1 cells and Caenorhabditis elegans and prevented obesity in high-fat diet-fed mice. Pharmacol. Res..

[12] Cai W.F., Yan M., Wang Z., Jiang M.P., Yan B., Shen C.Y. (2022). Optimization of the extract from flower of *Citrus aurantium* L. var. amara Engl. and its inhibition of lipid accumulation. J. Food Biochem..

[13] Boussaada O., Chemli R. (2006). Chemical composition of essential oils from flowers, leaves and peel of *Citrus aurantium* L. var. amara from Tunisia. J. Essent. Oil Bear. Plants.

[14] Wang X.S., Kapoor V., Smythe G.A. (2003). Extraction and chromatography-mass spectrometric analysis of the active principles from selected Chinese herbs and other medicinal plants. Am. J. Chin. Med..

[15] Sharifzadeh S., Karimi S., Abbasi H., Assari M. (2022). Sequential ultrasound-microwave technique as an efficient method for extraction of essential oil from Lavandula coronopifolia Poir. Food Meas. Charact..

[16] Mohagheghniapour A., Saharkhiz M.J., Golmakani M.T., Niakousari M. (2018). Variations in chemical compositions of essential oil from sour orange (*Citrus aurantium* L.) blossoms by different isolation methods. Sustain. Chem. Pharm..

[17] Bressanello D., Liberto E., Cordero C., Rubiolo P., Pellegrino G., Ruosi M.R., Bicchi C. (2017). Coffee aroma: Chemometric comparison of the chemical information provided by three different samplings combined with GC–MS to describe the sensory properties in cup. Food Chem..

[18] Cécile L., Céline C., Frédéric C. (2016). Fate and control of pathogenic and spoilage micro -organisms in orange blossom (*Citrus aurantium*) and rose flower (Rosa centifolia) hydrosols. J. Appl. Microbiol..

[19] Değirmenci H., Erkurt H. (2020). Relationship between volatile components, antimicrobial and antioxidant properties of the essential oil, hydrosol and extracts of *Citrus aurantium* L. flowers. Infect. Public Health.

[20] Su J., Pan Z.P., Xiao Y., Liang Z.E.N., Fu F.H. (2020). Study on the composition and the antioxidant activity of essential oil from flowers of *Citrus aurantium* L. var. amara Engl. Food Mach..

[21] Družić J., Jerković I., Marijanović Z., Roje M. (2016). Chemical biodiversity of the leaf and flower essential oils of *Citrus aurantium* L. from Dubrovnik area (Croatia) in comparison with *Citrus sinensis* L. Osbeck cv. Washington navel, *Citrus sinensis* L. Osbeck cv. Tarocco and *Citrus sinensis* L. Osbeck cv. Doppio Sanguigno. EssEntial Oil Res..

[22] Li S.S., Yang M., Wu W.J. (2022). Comparison of botanical traits and volatile constituents of grapefruit, lemon and *Citrus aurantium* ‘Daidai’. South. Chin. Fruit Trees.

[23] Lu X.M., Zhao C.Y., Shi H., Liao Y.C., Xu F., Du H.J., Xiao H., Zheng J.K. (2023). Nutrients and bioactives in citrus fruits: Different citrus varieties, fruit parts, and growth stages. Crit. Rev. Food Sci. Nutr..

[24] Addi M., Elbouzidi A., Abid M., Tungmunnithum D., Elamrani A., Hano C. (2021). An overview of bioactive flavonoids from citrus fruits. Appl. Sci..

[25] Pineau B., Barbe J.C., Leeuwen C.V., Dubourdieu D. (2009). Examples of Perceptive Interactions Involved in Specific “Red-” and “Black-berry” Aromas in Red Wines. J. Agric. Food Chem..

[26] Lv P., Zhong L., Jiang N., Jian S.H. (2018). Effect of different assisted extraction method on volatile compounds of the essential oil from *Citrus aurantium* L. var. amara Engl. Cereals Oils.

[27] Xiao Q.M., Zhang X.Y., Wang J., Lv Y.C. (2023). Comparative analysis on aromatic components of sun-dried green tea from different townships in Menghai county. Sci. Technol. Food Ind..

[28] Pereira I., Severino P., Santos A.C., Silva A.M., Souto E.B. (2018). Linalool bioactive properties and potential applicability in drug delivery systems. Colloids Surf. B Biointerfaces.

[29] Aprotosoaie A.C., Hăncianu M., Costache I.I., Miron A. (2014). Linalool: A review on a key odorant molecule with valuable biological properties. Flavour Fragr. J..

[30] Song S.Q., Tong Y.Z., Feng T., Zhu J.C., Wang Y.F., Sun M., Yao L.Y., Xu Z.M. (2017). Multidimensional analysis of odorous compounds in finger citron fruit (*Citrus medica* L. var. sarcodactylis Swingle) and identification of key aroma compounds. Food Sci..

[31] Chen Y., Zhang L.L., Wang W.Y., Wang G.G. (2023). Recent updates on bioactive properties of α-terpineol. J. Essent. Oil Res..

[32] Ma M.J., Su X.X., Tan X.Y., Niu Y., Li R.Y., Bian Q. (2022). Analysis of volatile flavor compounds in spicy sausages. Meat Res..

[33] Do Nascimento K.F., Moreira F.M.F., Alencar Santos J., Kassuya C.A.L., Croda J.H.R., Cardoso C.A.L., Vieira M.D.C., Góis Ruiz A.L.T., Ann Foglio M., De Carvalho J.E. (2018). Antioxidant, anti-inflammatory, antiproliferative and antimycobacterial activities of the essential oil of Psidium guineense Sw. and spathulenol. J. Ethnopharmacol..

[34] Li L., Jiang J.L., Hu J.L., Shi Y.X., Huang P., Ding D.K. (2023). Screening of key aroma components in essential oils from citrus peels of different cultivars. Sci. Technol. Food Ind..

[35] Celuppi L.C.M., Capelezzo A.P., Cima L.B., Zeferino R.C.F., Carniel T.A., Zanetti M., De Mello J.M.M., Fiori M.A., Riella H.G. (2023). Microbiological, thermal and mechanical performance of cellulose acetate films with geranyl acetate. Int. J. Biol. Macromol..

[36] Ping H.R., Wang Y.P., Chen J.N., Wang J.H. (2023). Analysis of taste and aroma differences between Miao and Shui ethnic red sour soup in Guizhou. Sci. Technol. Food Ind..

[37] Anandakumar P., Kamaraj S., Vanitha M.K. (2021). D-limonene: A multifunctional compound with potent therapeutic effects. J. Food Biochem..

[38] Xun Z.L., Ma X.H., Huang L.P., Wang M., Zhao Q.F. (2023). Analysis of berry aroma compounds from 47 table grape germplasm resources. J. Shanxi Agric. Univ. (Nat. Sci. Ed.).

[39] Silva B.O., Orlando J.B., Pires C.L., Hiruma-Lima C.A., De Mascarenhas Gaivao I., Perazzo F.F., Maistro E.L. (2021). Genotoxicity induced by nerol, an essential oil present in citric plants using human peripheral blood mononuclear cells (PBMC) and HepG_2_/C_3_A cells as a model. J. Toxicol. Environ. Health.

[40] Su L.D., Luo X.Y., Duan L.L., Xiong S.L. (2023). Optimization of processing technology and analysis of volatile flavor substances of garlic-flavor hot pot dipping sauce. China Condiment.

[41] Zhou X., Zhou J.S., Liu T.Y., Jiang L.W., Liu Y., Yin S.X., Rong Z.X., Chen H. (2023). Analysis of the effects in the Flavor Anchovy during circulating boiling brine based on HS-SPME-GC-MS. Sci. Technol. Food Ind..

[42] Almarzooqi S., Venkataraman B., Raj V., Alkuwaiti S.A.A., Das K.M., Collin P.D., Adrian T.E., Subramanya S.B. (2022). β-Myrcene mitigates colon inflammation by inhibiting MAP kinase and NF-κB signaling pathways. Molecules.

[43] Zhang Y.H., Chen X.Y., Wang C.Q., Jiao B.N. (2023). Difference analysis of volatile components in Orah mandarin (Citrus reticulata Blanco) from different areas. Food Ferment. Ind..

[44] Opdyke D.L.J. (1976). Neryl acetate. Food Cosmet. Toxicol..

[45] Gabriele A., Giovanna A., Vasileios B., De Lourdes Bastos M., Georges B., Andrew C., Cocconcelli P.S., Gerhard F., Boris K., Maryline K. (2016). Safety and efficacy of α, β-unsaturated straight-chain and branched-chain aliphatic primary alcohols, aldehydes, acids and esters belonging to chemical group 3 when used as flavourings for all animal species. Efsa J..

[46] Yin J., Jiang J., Liu W.J., Liao F., Hu A.F., Huang F.F., Liu J.L., Yang J. (2017). Combined GC-O and GC-MS identification of key aroma constituents in the essential oil of yangmei leaves. South China Fruits.

[47] García-Casas I., Montes A., De Los Santos D.M., Valor D., Pereyra C., De la Ossa E.M. (2023). Generation of high-porosity cerium oxide nanoparticles and their functionalization with caryophyllene oxide using supercritical carbon dioxide. J. Supercrit. Fluids.

[48] Zhou Q.Y., Zeng W.X., Zhang J.Z., Zeng J.L., Zhang X.M., Zeng L.K., Ling C.J. (2023). Analysis on quality components and aroma characteristics of Hakka roasted green tea. Mod. Food Sci. Technol..

[49] Deng X.Y., Xue J.S., Li H.Y., Ma Z.Q., Fu Q., Qu R., Ma S.P. (2015). Geraniol produces antidepressant-like effects in a chronic unpredictable mild stress mice model. Physiol. Behav..

[50] Guo X.Y., Ho C.T., Schwab W., Wan X.C. (2021). Effect of the roasting degree on flavor quality of large-leaf yellow tea. Food Chem..

[51] Hu Z.Y., Liu W., He S., Hu X.Q., Zhang J.H., Shan Y. (2021). Analysis of volatile components in three varieties of kumquat by headspace solid phase microextraction-gas chromatography-mass spectrometry. Food Sci..

[52] Wu Q.H., Jiang W., Yang J.Y., Si X.X., Yi A., Wang M.J., Zhao Y., Shan S.Y., Zhang F.M. (2023). Effect of solvent extraction on the key aroma components of Tamarindus indica L. pulp. J. Food Compos. Anal..

[53] Zeng L., Fu Y.Q., Huang J.S., Wang J.R., Jin S., Yin J.F., Xu Y.Q. (2022). Comparative analysis of volatile compounds in Tieguanyin with different types based on HS–SPME–GC–MS. Foods.

